# Retraction: Influence of organic manures on soil nutrient content, microbial population, yield and quality parameters of pomegranate (*Punica granatum* L.) cv. Bhagwa

**DOI:** 10.1371/journal.pone.0273488

**Published:** 2022-08-31

**Authors:** The PLOS ONE Editors

The *PLOS ONE* Editors retract this article [1] because it was identified as one of a series of submissions for which we have concerns about authorship, competing interests, and peer review. We regret that the issues were not addressed prior to the article’s publication.

NRA, TJ, MA, UK, RCC, HLB, PS, LNM, SKS, and RR did not agree with retraction. KL, MMH, and AAAS either did not reply or could not be reached.
